# Assessment of chemotherapy regimens on radiation pneumonitis in patients with unresectable stage III non‐small‐cell lung cancer after definitive chemoradiotherapy

**DOI:** 10.1111/1759-7714.14005

**Published:** 2021-05-18

**Authors:** Tadashi Sakaguchi, Kentaro Ito, Naoki Furuya, Kei Morikawa, Kentaro Fujiwara, Yoichi Nishii, Takeo Inoue, Osamu Hataji, Masamichi Mineshita

**Affiliations:** ^1^ Division of Respiratory Medicine, Department of Internal Medicine St. Marianna University School of Medicine Kawasaki Japan; ^2^ Respiratory Center Matsusaka Municipal Hospital Matsusaka Japan

**Keywords:** chemoradiotherapy, consolidation therapy, durvalumab, non‐small cell lung cancer, radiation pneumonitis

## Abstract

**Background:**

Consolidation therapy with durvalumab after concurrent chemoradiotherapy has been reported to significantly prolong progression‐free survival and overall survival in patients with stage III unresectable non‐small cell lung cancer (NSCLC). However, which chemotherapy regimen should be selected for consolidation therapy with durvalumab is currently unknown.

**Methods:**

We retrospectively reviewed consecutive patients with unresectable stage III NSCLC who received concurrent definitive chemoradiotherapy with platinum‐based chemotherapy. We reviewed the timing and severity of radiation pneumonitis by assessing chemotherapy regimens and histology.

**Results:**

A total of 103 patients were identified. Fourteen patients (13.6%) developed grade 2 or greater radiation pneumonitis within 42 days after chemoradiotherapy. No adenocarcinoma patients treated with a regimen of cisplatin plus pemetrexed developed grade 2 or greater radiation pneumonitis within 42 days; however, 20% of patients who were treated with carboplatin plus paclitaxel developed grade 2 or greater radiation pneumonitis. Furthermore, the objective response rates and disease control rates of cisplatin plus pemetrexed were equal to or greater than those of carboplatin plus paclitaxel in adenocarcinoma patients.

**Conclusion:**

Cisplatin plus pemetrexed regimen may be a preferable option to consider for subsequent consolidation therapy with durvalumab in patients with unresectable stage III adenocarcinoma.

## Graphical abstract

Among a total of 103 patients with unresectable stage III NSCLC who received concurrent definitive chemoradiotherapy, 13.6% of patients developed grade 2 or greater radiation pneumonitis within 42 days after chemoradiotherapy. No adenocarcinoma patients treated with cisplatin plus pemetrexed developed grade 2 or greater radiation pneumonitis within 42 days; however, 20% of patients treated with carboplatin plus paclitaxel developed grade 2 or greater radiation pneumonitis.

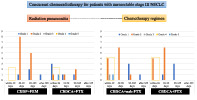

## INTRODUCTION

Approximately one‐third of patients with non‐small‐cell lung cancer (NSCLC) present with stage III locally advanced nonmetastatic disease.^1^ Until recently, the standard of care for patients with unresectable stage III NSCLC was definitive chemoradiotherapy.^2^ However, the PACIFIC trial showed that durvalumab significantly prolonged progression‐free survival (PFS) and overall survival (OS) when compared with placebo in patients with unresectable stage III NSCLC without disease progression after definitive chemoradiotherapy.^3^, ^4^ Therefore, consolidation therapy using durvalumab after definitive chemoradiotherapy has now become standard therapy in patients with unresectable stage III NSCLC.

In clinical practice, it was estimated that ~23%–30% of patients with unresectable stage III NSCLC treated with definitive chemoradiotherapy might be ineligible to receive consolidation therapy with durvalumab under the same conditions as the PACIFIC trial.^5^, ^6^ Early onset of pneumonitis, especially radiation pneumonitis, is one of the major limiting factors for the administration of durvalumab as a consolidation therapy.

Although most patients receiving thoracic irradiation are at risk for radiation pneumonitis, several factors categorized as radiation risk factors, disease risk factors, and host risk factors, may modify their pneumonitis risk.^7^ Although the incidence and timing of radiation pneumonitis might be different between each chemotherapy regimen, there are few previous reports that investigate this clinical question.^8^, ^9^ The PACIFIC trial was designed to initiate durvalumab within 42 days after completion of chemoradiotherapy. Therefore, regimens with lower risks of symptomatic radiation pneumonitis within the early period are considered preferable when deciding on definitive chemoradiotherapy for unresectable stage III NSCLC patients.

From this perspective, we retrospectively evaluated the timing and severity of radiation pneumonitis based on chemotherapy regimens used for definitive chemoradiotherapy in patients with unresectable stage III NSCLC.

## METHODS

### Patient selection

This retrospective study was conducted at St. Marianna University School of Medicine and Matsusaka Municipal Hospital, Japan. We reviewed the electronic data of consecutive patients with unresectable stage III NSCLC who received concurrent definitive chemoradiotherapy with platinum‐based chemotherapy from January 2011 to May 2018. Patients who were treated with sequential chemoradiotherapy, non‐platinum regimens, or as adjuvant or neoadjuvant chemoradiotherapy were excluded. Thoracic radiation therapy (TRT) was planned with normal tissue dose‐volume constraints, such as the percent volume of the lung receiving at least 20 Gy (V20) ≤35%, the percent volume of the lung receiving at least 5 Gy (V5) ≤70%, and the mean lung dose (MLD) ≤20 Gy. Clinical data assessments included patient characteristics, pathological findings, chemotherapy regimens, lung irradiation dose, the incidence, onset and severity of radiation pneumonitis, treatments and clinical course for radiation pneumonitis, and chemoradiotherapy response. Data cut off was July 2018, 1 month before the approval of durvalumab in Japan. This study protocol was approved by the relevant ethics committees.

### Outcome assessments

Primary outcomes of this study were the incidence rate, time to onset, and severity of radiation pneumonitis based on chemotherapy regimens. Radiation pneumonitis was diagnosed by the discretion of the attending doctor based on symptoms, the timing of pneumonitis development during or after chemoradiotherapy, the relationship between the extent of pneumonitis and the irradiation field, and by clinically excluding other causes including drug‐induced pneumonitis. Chest computed tomography (CT) and chest X‐ray images were used for the diagnosis of radiation pneumonitis. The time to onset for radiation pneumonitis was defined as the period from the completion or discontinuation of chemoradiotherapy to when pneumonitis was initially documented. The severity of radiation pneumonitis was graded according to the National Cancer Institute Common Terminology Criteria for Adverse Events, version 4.03. Further, we evaluated the rate of grade 2 or greater radiation pneumonitis within 42 days after chemoradiotherapy completion, which is in accordance with the criteria of the PACIFIC trial.^3^, ^4^ Secondary outcomes were the objective response rate (ORR) and disease control rate (DCR) for each treatment regimen. ORR was defined as the proportion of patients with the best overall response for complete response (CR) or partial response (PR). DCR was defined as the proportion of patients with the best overall response for CR, PR, or stable disease (SD). The Response Evaluation Criteria in Solid Tumors, Version 1.1 was used to assess collected data.^10^ We evaluated the treatment and clinical course for radiation pneumonitis as post hoc analysis.

### Statistical analysis

Time to onset of radiation pneumonitis was estimated using the Kaplan–Meier method and compared using the log‐rank test. Statistical analyses were performed using Student's *t*‐test for continuous variables, and χ^2^ test and Fisher's exact test for categorical variables. Statistical analyses were performed using SPSS software, version 23.0 (SPSS). A *p*‐value <0.05 was considered statistically significant.

## RESULTS

### Patient characteristics

A total of 103 patients were identified for analysis. Patient characteristics are shown in Table 1. The majority of the patients were male (86%), with a median age of 68 years (range, 49–85 years). Over half (52%) of tumor histology findings were squamous cell carcinoma, followed by adenocarcinoma (33%). The lung irradiation dose most patients received was between 60 and 66 Gy. For the 34 patients with adenocarcinoma, 19 (56%) received cisplatin plus pemetrexed, whereas 10 (29%) received carboplatin plus paclitaxel regimens. For the 54 patients with squamous cell carcinoma, 25 (46%) received carboplatin plus paclitaxel, whereas 22 (41%) received carboplatin plus nab‐paclitaxel regimens.

**TABLE 1:** Patient characteristics

Characteristics	*n* = 103	(%)
Median age	68	
Range	49–85	
Sex		
Female	14	14
ECOG PS		
0–1	98	95
2–3	5	5
Smoking history		
Never	9	9
Former	57	55
Current	36	35
Unknown	1	1
cStage (UICC‐8)		
III A	59	57
III B	37	36
III C	7	7
Histology		
ADC	34	33
SCC	54	52
Other	15	15
Radiotherapy technique		
3D‐CRT	103	100%
Radiotherapy dose		
60 Gy	35	34
63 Gy	47	45
66 Gy	15	15
Other	6	6
Regimen		
CDDP base		
+PEM	21	20
+DTX	6	6
+ETP	3	3
+VNR	2	2
CBDCA base		
+nab‐PTX	29	28
+PTX	37	36
+PEM	3	3
+ETP	1	1
+S‐1	1	1

Abbreviations: 3D‐CRT, three‐dimensional conformal external beam radiation therapy; ADC, adenocarcinoma; CBDCA, carboplatin; CDDP, cisplatin; DTX, docetaxel; ECOG, Eastern Cooperative Oncology Group; ETP, etoposide; nab‐PTX, nanoparticle albumin‐bound paclitaxel; PEM, pemetrexed; PS, performance status; PTX, paclitaxel; SCC, squamous cell carcinoma; UICC, Union for International Cancer Control; VNR, vinorelbine.

### Radiation pneumonitis

**FIGURE 1:**
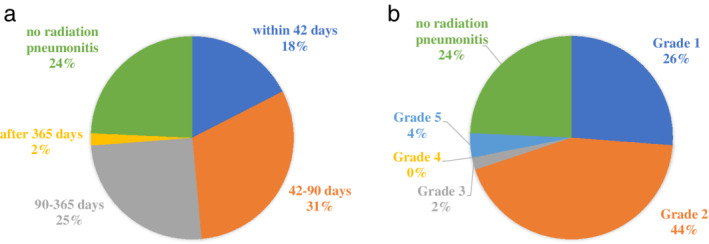
Onset and severity of radiation pneumonitis. (a) Proportion based on the onset after chemoradiotherapy completion or discontinuation. (b) Proportion based on the severity of radiation pneumonitis

**FIGURE 2:**
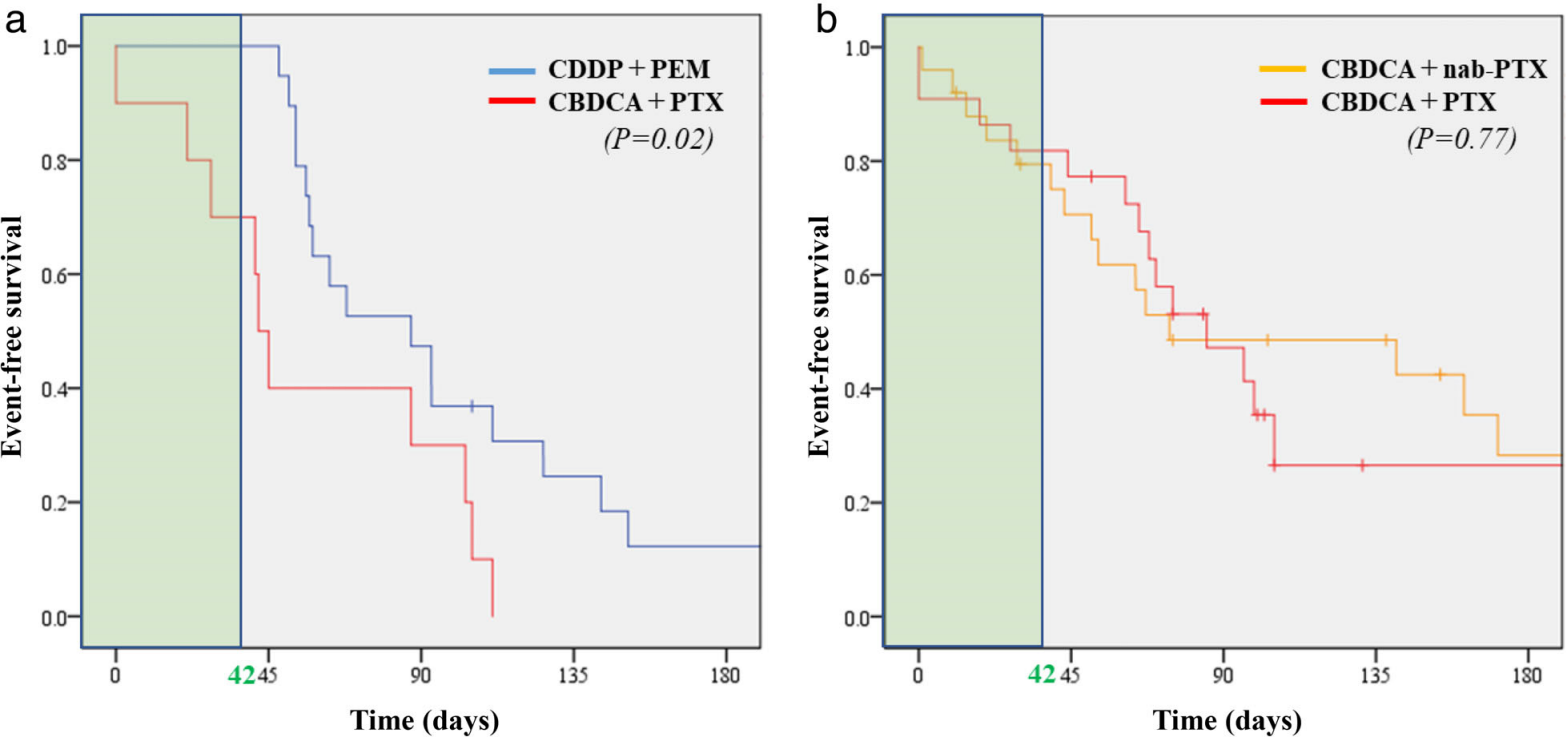
Event‐free survival of time to onset for radiation pneumonitis according to histology and chemotherapy regimen. (a) Adenocarcinoma. (b) Squamous‐cell carcinoma. Square portions in green show radiation pneumonitis at early onset

**FIGURE 3:**
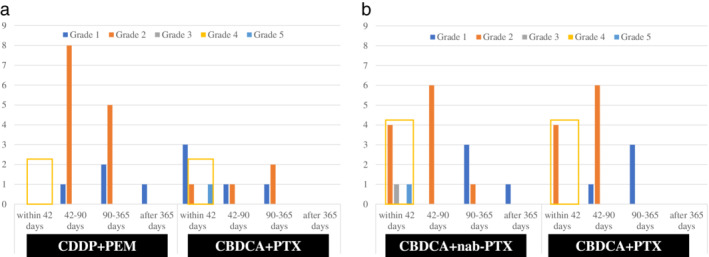
Severity and onset for radiation pneumonitis according to histology and chemotherapy regimen. (a) Adenocarcinoma. (b) Squamous cell carcinoma. The orange box highlights patients who developed radiation pneumonitis of grade 2 or greater at early onset and could be unable to meet the criteria for durvalumab

The incidence rate for radiation pneumonitis of any grade occurred in 78 patients (76%) receiving definitive chemoradiotherapy (Figure 1(a),(b)). The incidence of grades 1 or 2 radiation pneumonitis was 70%, whereas grade 3 or greater was 6% (Figure 1(b)). Radiation pneumonitis at early onset, which was defined as pneumonitis within 42 days after completion or discontinuation of chemoradiotherapy as per criteria from the PACIFIC trial, developed in 18 patients (18%). Of these 14 (14%) developed grade 2 or greater radiation pneumonitis. The time to onset of radiation pneumonitis based on histology and frequency of regimens for each histology type are shown in Figure 2. The incidence of radiation pneumonitis and radiation pneumonitis at early onset with grade 2 or greater based on regimen frequency for each common histology type are shown in Table 2 and Figure 3. The characteristics of these subgroups are shown in Table S1. Among patients with adenocarcinoma, those treated with a regimen of cisplatin plus pemetrexed did not develop into radiation pneumonitis at early onset with grade 2 or greater, unlike those treated with carboplatin plus paclitaxel. Among squamous cell carcinoma, patients treated with carboplatin plus paclitaxel and carboplatin plus nab‐paclitaxel equally developed into radiation pneumonitis at early onset with grade 2 or greater.

**TABLE 2:** Radiation pneumonitis in early onset with grade 2 or greater based on regimen frequency

	ADC			SCC		
	CDDP + PEM (*n* = 19)	CBDCA + PTX (*n* = 10)	*p*‐value	CBDCA + PTX (*n* = 25)	CBDCA + nab‐PTX (*n* = 22)	*p*‐value
Incidence rate of RP						
Total	89%	100%	0.53	68%	64%	0.77
Early onset with grade 2 or greater	0%	20%	0.11	24%	18%	0.73

Abbreviations: ADC, adenocarcinoma; CBDCA, carboplatin; CDDP, cisplatin; nab‐PTX, nanoparticle albumin‐bound paclitaxel; PEM, pemetrexed; PTX, paclitaxel; SCC, squamous cell carcinoma.

### Response of chemoradiotherapy by regimen

The ORR and DCR of concurrent definitive chemoradiotherapy according to the frequency of regimens for each histology are shown in Table 3. ORR and DCR for cisplatin plus pemetrexed were equal or greater than those for carboplatin plus paclitaxel in adenocarcinoma patients. ORR and DCR for carboplatin plus nab‐paclitaxel were equal to those of carboplatin plus paclitaxel for squamous cell carcinoma.

**TABLE 3:** Overall response based on regimen frequency for each major histology

	ADC		SCC	
	CDDP + PEM (*n* = 19)	CBDCA + PTX (*n* = 10)	CBDCA + nab‐PTX (*n* = 25)	CBDCA + PTX (*n* = 22)
CR	1 (5%)	0 (0%)	2 (8%)	1 (5%)
PR	14 (74%)	7 (70%)	15 (60%)	15 (68%)
SD	4 (21%)	2 (20%)	5 (20%)	4 (18%)
PD	0 (0%)	1 (10%)	1 (4%)	2 (9%)
NE	0 (0%)	0 (0%)	2 (8%)	0 (0%)
ORR	79%	70%	68%	73%
DCR	100%	90%	88%	91%

Abbreviations: ADC, adenocarcinoma; CBDCA, carboplatin; CDDP, cisplatin; CR, complete response; DCR, disease control rate; nab‐PTX, nanoparticle albumin‐bound paclitaxel; NE, not evaluated; ORR, overall response rate; PD, progressive disease; PEM, pemetrexed; PR, partial response; PTX, paclitaxel; SCC, squamous cell carcinoma; SD, stable disease.

### Clinical course for radiation pneumonitis

Of the 78 patients with radiation pneumonitis, 49 (63%) received steroid treatment. Of these, 37 patients (47%) tapered steroid use of prednisolone equivalent to 10 mg or less within 12 weeks after onset of radiation pneumonitis. The median time to taper steroid use to 10 mg or less was 53 days. The median time of steroid therapy termination from the initiation of radiation pneumonitis was 108 days. A total of four patients (4%) had grade 5 radiation pneumonitis, all received platinum plus taxane.

## DISCUSSION

To the best of our knowledge, this is the first report to focus on the timing and severity of radiation pneumonitis for consideration of subsequent durvalumab therapy based on chemotherapy regimens in unresectable stage III NSCLC patients treated with concurrent chemoradiotherapy. The two most frequent chemotherapy regimens used in the PACIFIC trial were carboplatin plus paclitaxel and cisplatin plus etoposide.^3^, ^4^ Previous reports showed chemotherapy was a predictive factor for radiation pneumonitis.^11^, ^12^ Furthermore, it was reported that various drugs when administered were associated with an increased risk of radiation pneumonitis, which included cytotoxic agents such as bleomycin, cyclophosphamide, vincristine, taxanes, doxorubicin, dactinomycin, mitomycin, gemcitabine, and targeted agents like erlotinib and bevacizumab.^13^ For chemotherapy regimens frequently used worldwide, one prospective phase II study showed that the incidence of grade 2 or greater radiation pneumonitis was more frequent in the carboplatin plus paclitaxel arm than the cisplatin plus etoposide arm (48.5% vs. 25%, *p* = 0.06),^14^ and one systematic review reported that carboplatin plus paclitaxel was a high risk factor for symptomatic radiation pneumonitis relative to cisplatin plus etoposide.^8^ Therefore, the use of carboplatin plus paclitaxel might make it difficult to deliver subsequent consolidation therapy with durvalumab because of radiation pneumonitis.

For regimens using pemetrexed, a review of concurrent pemetrexed and radiation therapy in patients with inoperable stage III NSCLC including six phase II trials, reported the rates of radiation pneumonitis ranged between 0% and 23%, with promising activity and an acceptable toxicity profile.^15^ The phase III PROCLAIM study evaluated OS for concurrent cisplatin plus pemetrexed and TRT followed by consolidation of pemetrexed, versus cisplatin plus etoposide and TRT followed by non‐pemetrexed consolidation therapy in locally advanced non‐squamous NSCLC.^16^ This study showed no incidences of grade 3 or 4 pneumonitis in the concurrent phase for the arm with three cycles of cisplatin plus pemetrexed, whereas two patients (0.7%) developed into grade 3 or 4 pneumonitis in concurrent phase for the arm with two cycles of cisplatin plus etoposide. In our study, no patients in the adenocarcinoma cohort treated with cisplatin plus pemetrexed developed into grade 2 or greater radiation pneumonitis at early onset, whereas 2 patients treated with carboplatin plus paclitaxel developed grade 2 or greater radiation pneumonitis. Our results suggest that cisplatin plus pemetrexed is less likely to develop into early onset radiation pneumonitis and may be favorable for subsequent consolidation therapy with durvalumab compared with carboplatin plus paclitaxel for adenocarcinoma patients. Furthermore, the DCR of cisplatin plus pemetrexed was equal or better than carboplatin plus paclitaxel in our study. To deliver consolidation therapy with durvalumab, the overall response at completion of concurrent chemoradiotherapy should be SD or better. Therefore, cisplatin plus pemetrexed may be preferable for subsequent consolidation therapy with durvalumab in terms of efficacy. For squamous cell carcinoma, the incidence rate of grade 2 or greater radiation pneumonitis at early onset and DCR of carboplatin plus paclitaxel and carboplatin plus nab‐paclitaxel were equal.

There were several limitations to this study. First, this was a small retrospective study and patients did not receive scheduled image assessment. Further evaluation with larger cohorts including periodic imaging assessment is required. However, to evaluate the natural course of radiation pneumonitis after definitive chemoradiotherapy prospectively would be inappropriate because subsequent consolidation therapy with durvalumab has been established as a standard therapy for patients with unresectable stage III NSCLC. Second, the diagnosis of radiation pneumonitis was at the discretion of the attending doctors and therefore, the reliability of the diagnosis, that is, whether it was properly differentiated from other pneumonitis may be uncertain. Third, we were unable to evaluate some radiotherapeutic predictive factors, such as V5, V20, and MLD, which are generally considered as important predictive factors for radiation pneumonitis. However, these predictive factors would be difficult to calculate because each patient's therapeutic strategy varied depending on their radiologist. Fourth, other risk factors such as pulmonary comorbidities may have modified the timing and severity of radiation pneumonitis in this study. Although patients with interstitial lung disease were rarely included, patients with emphysema were included. However, it is considered controversial as to whether the presence and severity of emphysema has a positive or negative risk factor for radiation pneumonitis,^17^, ^18^, ^19^ and the smoking histories of subgroups based on regimen frequency for each common histology type are not significantly different in our study. Finally, this study evaluated only a few selected regimens; therefore, in the future, various regimens should be evaluated to select which chemoradiotherapies are favorable for consolidation therapy with durvalumab.

## CONCLUSION

When deciding on chemotherapy regimens of definitive chemoradiotherapy for unresectable stage III NSCLC patients, we should consider feasibility of consolidation therapy with durvalumab. Cisplatin plus pemetrexed may be an option to consider for subsequent consolidation therapy with durvalumab in patients with adenocarcinoma.

## CONFLICT OF INTEREST

Matsusaka Municipal Hospital has received research grant funding from Novartis, GlaxoSmithKline, AstraZeneca, Daiichi Sankyo, Bayer, and Boehringer Ingelheim. K. Ito has received speaker fees as honoraria from Eli Lilly Japan, Chugai, AstraZeneca, MSD, Boehringer Ingelheim Japan, Ono, and Pfizer Japan. N. Furuya has received speaker fees as honoraria from Eli Lilly Japan, Chugai, AstraZeneca, Bristol Myers Squibb, Taiho, Boehringer Ingelheim Japan, Ono, and Pfizer Japan. O. Hataji received speaker fees as honoraria from Novartis Pharma, AstraZeneca, and Boehringer Ingelheim Japan. M. Mineshita received honoraria from Eli Lilly Japan, Boehringer Ingelheim Japan, AstraZeneca, Bristol Myers Squibb, MSD, and Novartis Pharma.

## Supporting information


**Table S1** Characteristics of patients based on regimen frequency for each histology type
