# Supplementary material for: Assessment of chemotherapy regimens on radiation pneumonitis in patients with unresectable stage III non‐small‐cell lung cancer after definitive chemoradiotherapy
Source: Thorac Cancer. 2021 May 18;12(13):2024–30. doi: 10.1111/1759-7714.14005 (PMC8258360; doi:10.1111/1759-7714.14005)
Supplement: Supplementary file 1 — Table S1 Characteristics of patients based on regimen frequency for each histology type [file TCA-12-2024-s001.docx]

Supplemental table 1. Characteristics of patients based on regimen frequency for each histology type

|  | ADC | | | SCC | | |
| --- | --- | --- | --- | --- | --- | --- |
|  | CDDP＋PEM  (N=19) | CBDCA＋PTX (N=10) | P-value | CBDCA＋nab-PTX (N=25) | CBDCA＋PTX (N=22) | P-value |
| Median age | 65 | 70.5 | <0.05 | 72 | 70 | 0.6 |
| Range | 53-72 | 57-76 |  | 54-85 | 51-84 |  |
| Sex |  |  | 0.19 |  |  | 0.21 |
| Female | 3 (16%) | 4 (40%) |  | 0 (0%) | 2 (9%) |  |
| ECOG PS |  |  | NA |  |  | 0.61 |
| 0-1 | 19 (100%) | 10 (100%) |  | 22 (88%) | 21 (95%) |  |
| 2-3 | 0 (0%) | 0 (0%) |  | 3 (12%) | 1 (5%) |  |
| Smoking history |  |  | 0.64 |  |  | 0.06 |
| Never | 2 (10%) | 2 (20%) |  | 0 (0%) | 2 (9%) |  |
| Former | 10 (53%) | 6 (60%) |  | 12 (50%) | 15 (68%) |  |
| Current | 7 (37%) | 2 (20%) |  | 12 (50%) | 5 (23%) |  |
| cStage (UICC-8) |  |  | 0.47 |  |  | 0.68 |
| III A | 13 (68%) | 7 (70%) |  | 13 (52%) | 14 (64%) |  |
| III B | 6 (32%) | 2 (20%) |  | 9 (36%) | 7 (32%) |  |
| III C | 0 (0%) | 1 (10%) |  | 3 (12%) | 1 (5%) |  |
| Radiotherapy dose |  |  | NA |  |  | 1 |
| 60-66 Gy | 19 (100%) | 10 (100%) |  | 24 (96%) | 21 (95%) |  |
| Other | 0 (0%) | 0 (0%) |  | 1 (4%) | 1 (5%) |  |

Abbreviations: ECOG, Eastern Cooperative Oncology Group; PS, performance status; UICC, Union for International Cancer Control; ADC, adenocarcinoma; SCC, squamous cell carcinoma; CDDP, cisplatin; PEM, pemetrexed; CBDCA, carboplatin; PTX, paclitaxel; nab-PTX, nanoparticle albumin-bound paclitaxel; NA, not available.
